# Computational strategies for alternative single-step Bayesian regression models with large numbers of genotyped and non-genotyped animals

**DOI:** 10.1186/s12711-016-0273-2

**Published:** 2016-12-08

**Authors:** Rohan L. Fernando, Hao Cheng, Bruce L. Golden, Dorian J. Garrick

**Affiliations:** 1 Department of Animal Science, Iowa State University, Ames, IA 50011 USA; 2 Theta Solutions, LLC, Atascadero, CA 93422 USA; 3 Institute of Veterinary, Animal and Biomedical Sciences, Massey University, Palmerston North, New Zealand

## Abstract

**Background:**

Two types of models have been used for single-step genomic prediction and genome-wide association studies that include phenotypes from both genotyped animals and their non-genotyped relatives. The two types are breeding value models (BVM) that fit breeding values explicitly and marker effects models (MEM) that express the breeding values in terms of the effects of observed or imputed genotypes. MEM can accommodate a wider class of analyses, including variable selection or mixture model analyses. The order of the equations that need to be solved and the inverses required in their construction vary widely, and thus the computational effort required depends upon the size of the pedigree, the number of genotyped animals and the number of loci.

**Theory:**

We present computational strategies to avoid storing large, dense blocks of the MME that involve imputed genotypes. Furthermore, we present a hybrid model that fits a MEM for animals with observed genotypes and a BVM for those without genotypes. The hybrid model is computationally attractive for pedigree files containing millions of animals with a large proportion of those being genotyped.

**Application:**

We demonstrate the practicality on both the original MEM and the hybrid model using real data with 6,179,960 animals in the pedigree with 4,934,101 phenotypes and 31,453 animals genotyped at 40,214 informative loci. To complete a single-trait analysis on a desk-top computer with four graphics cards required about 3 h using the hybrid model to obtain both preconditioned conjugate gradient solutions and 42,000 Markov chain Monte-Carlo (MCMC) samples of breeding values, which allowed making inferences from posterior means, variances and covariances. The MCMC sampling required one quarter of the effort when the hybrid model was used compared to the published MEM.

**Conclusions:**

We present a hybrid model that fits a MEM for animals with genotypes and a BVM for those without genotypes. Its practicality and considerable reduction in computing effort was demonstrated. This model can readily be extended to accommodate multiple traits, multiple breeds, maternal effects, and additional random effects such as polygenic residual effects.

## Background

Two types of equivalent mixed linear models are used for whole-genome analyses in livestock [1]. The first type, which we refer to as marker effects models (MEM), includes random effects ($\varvec{\alpha }$) of marker genotype covariates (${\mathbf {M}}_{g}$) in the model [2, 3]. The second type, which we refer to as breeding value models (BVM), includes the breeding values of the animals, ${\mathbf {u}}_{g}={\mathbf {M}}_{g}\varvec{\alpha }$, as a random effect that has a covariance computed from ${\mathbf {M}}_g$ [1, 2, 4–6] rather than from the pedigree.

It was shown that the BVM can be adapted for what is known as single-step genomic best linear unbiased prediction (SS-GBLUP) that combines information from animals with genotypes and from those without genotypes in a single BLUP analysis [7–9]. However, the SS-GBLUP analysis requires computing the inverse of ${\mathbf {G}}$, which is the matrix of genomic relationships of the animals with genotypes [8, 9]. When the number $N_{g}$ of genotyped animals exceeds the number of markers, ${\mathbf {G}}$ is singular, but a full-rank matrix such as ${\mathbf {G}}^{*}=0.95{\mathbf {G}}+0.05{\mathbf {A}},$ with ${\mathbf {A}}$ being the pedigree-based relationship matrix might be used in its place. Single-step analyses based on the MEM do not require computing ${\mathbf {G}}$ or its inverse [10]. Furthermore, Bayesian regression analyses based on the MEM are not limited to assuming a normal prior for $\varvec{\alpha }$, which is implicit in SS-GBLUP; Bayesian regression models can accommodate various priors including the *t* distribution as in BayesA [3, 11], the double exponential distribution as in Bayesian LASSO [12] or mixtures of the *t* distribution or the normal distribution [3, 11, 13] as in BayesB or BayesC. However, the MME that correspond to single-step MEM (SS-MEM) types of models contain dense blocks that correspond to the imputed genotypes of animals with missing genotypes [10], and those blocks can be large if many animals have missing genotypes.

Liu et al. [14] developed a single-step method based on the BVM with direct estimation of marker effects (SSME-GBLUP). An advantage of that method over SS-GBLUP is that it does not require computing ${\mathbf {G}}$ or its inverse. Also, their method can be used for Bayesian regression models [14]. However, the MME for SSME-GBLUP contains expressions that involve the inverse of the pedigree-based relationship matrix, ${\mathbf {A}}_{gg},$ for the animals with genotypes. This is a dense matrix, and therefore a computational strategy was proposed to avoid computing its inverse but it requires solving a dense system of equations of order $N_{g}$ within each round of Jacobi or pre-conditioned conjugate gradient (PCG) iteration for solution of the MME or within each round of MCMC sampling for Bayesian inference with models such as BayesA or BayesB [3]. Equation (A1) in Legarra and Ducrocq [15] also present a set of similar MME with marker effects for genotyped animals and breeding values for non-genotyped animals. As with the MME in Liu et al. [14], the advantage of the MME of Legarra and Ducrocq [15] is that they do not require the computation of ${\mathbf {G}}$ or its inverse but require computing the inverse of ${\mathbf {A}}_{gg}$. Recently, in some livestock such as dairy cattle, $N_{g}$ has increased towards a million or more, and thus, solving a dense system of equations of order $N_{g}$ within each round of iteration will place a heavy burden on SSME-GBLUP in computing time and storage requirements.

The objective of this paper is to present computational strategies for whole-genome analyses based on the SS-MEM that avoid storing large, dense blocks of the MME that involve imputed genotypes. First, we will show this for the MME given in [10]. Second, we will present what we refer to as a hybrid type model (HM) that uses a MEM for the animals with marker genotypes and a BVM for animals without genotypes. The MME that correspond to this model also has dense blocks that correspond to animals with missing genotypes. However, in Bayesian regression analyses based on this hybrid model, storing the dense blocks can be avoided even more efficiently than was the case for the MME given in [10]. Finally, we will present the computer storage and time required for a real application.

## Theory

In most genomic analyses, the columns of the matrix ${\mathbf {M}}_{g}$ of marker covariates are centered to have zero expectations. This ensures that the vector of breeding values, ${\mathbf {u}}_{g}={\mathbf {M}}_{g}\varvec{\alpha }$, has a mean of ${\mathbf {0}}$. Centering ${\mathbf {M}}_{g}$ requires knowing the expected value of the marker covariates for founder animals. Often, these expected values are unknown, but can be incorporated into the model as a location parameter [10, 16]. However, to simplify our presentation without loss of generality we assume that ${\mathbf {M}}_{g}$ is a matrix of correctly centered marker covariates.

### Marker effects model for single-step Bayesian regression

As in Fernando et al. [10], a MEM for single-step Bayesian regression analyses can be derived from writing the model equation as:

1 $$\begin{aligned} \mathbf {\left[ \begin{array}{c} {\mathbf {y}}_{n}\\ {\mathbf {y}}_{g} \end{array}\right] }=\left[ \begin{array}{c} {\mathbf {X}}_{n}\\ {\mathbf {X}}_{g} \end{array}\right] \varvec{\beta }+\left[ \begin{array}{cc} {\mathbf {Z}}_{n} &{} {\mathbf {0}}\\ {\mathbf {0}} &{} {\mathbf {Z}}_{g} \end{array}\right] \left[ \begin{array}{l} {\mathbf {M}}_{n}\varvec{\alpha }+\varvec{\epsilon }\\ {\mathbf {M}}_{g}\varvec{\alpha } \end{array}\right] +{\mathbf {e}}, \end{aligned}$$

where the vectors and matrices for animals without genotypes are denoted with a subscript *n* and those for the animals with genotypes with a subscript *g*. Thus, ${\mathbf {y}}_{n}$ and ${\mathbf {y}}_{g}$ are the vectors of phenotypic values, ${\mathbf {X}}_{n}$ and ${\mathbf {X}}_{g}$ are the incidence matrices for the fixed effects, $\varvec{\beta }$, ${\mathbf {Z}}_{n}$ and ${\mathbf {Z}}_{g}$ are incidence matrices that relate the breeding values of animals, $$\left[ \begin{array}{l} {\mathbf {M}}_{n}\varvec{\alpha }+\varvec{\epsilon }\\ {\mathbf {M}}_{g}\varvec{\alpha } \end{array}\right]$$, to the phenotypic values, ${\mathbf {M}}_{g}$ is the matrix of centered marker covariates for animals with genotypes, ${\mathbf {M}}_{n}={\mathbf {A}}_{ng}{\mathbf {A}}_{gg}^{-1}{\mathbf {M}}_{g}$, is the matrix of imputed marker covariates for animals with missing genotypes, $\varvec{\alpha }$ is the vector of random marker effects, $\varvec{\epsilon }$ is the vector of imputation residuals with null means and covariance matrix proportional to the inverse of ${\mathbf {A}}^{nn}$, the sub-matrix corresponding to animals with missing genotypes in the inverse of the matrix ${\mathbf {A}}$ of pedigree-based additive relationships, and ${\mathbf {e}}$ is a vector of residuals. The matrix of imputed genotypes can be more efficiently computed by solving the sparse system of equations [10]:

2 $$\begin{aligned} {\mathbf {A}}^{nn} {\mathbf {M}}_{n}=- {\mathbf {A}}^{ng} {\mathbf {M}}_{g}. \end{aligned}$$

Depending on the prior used for $\varvec{\alpha }$, Model (1) can be used for a range of single-step Bayesian regression analyses, including single-step BLUP, BayesA, BayesB, BayesC or Bayesian LASSO [10]. Those models (1) and their corresponding analyses assume that the breeding values can be adequately explained by the marker covariates. If that assumption does not hold, a polygenic residual with a mean of zero and a covariance matrix that is proportional to **A **can be included as an additional effect in the model.

The MME that correspond to Model (1) for BayesC with $\pi =0$ are:

3 $$\begin{aligned} \left[ \begin{array}{lll} {\mathbf {X}}'{\mathbf {X}} &{} {\mathbf {X}}'{\mathbf {ZM}} &{} {\mathbf {X}}_{n}'{\mathbf {Z}}_{n}\\ {\mathbf {M}}'{\mathbf {Z}}' {\mathbf {X}} &{} {\mathbf {M}}' {\mathbf {Z}}' {\mathbf {ZM}}+ \mathbf {I\dfrac{\sigma _{e}^{2}}{\sigma _{\alpha }^{2}}} &{} {\mathbf {M}}_{n}'{\mathbf {Z}}'_{n}{\mathbf {Z}}_{n}\\ {\mathbf {Z}}'_{n} {\mathbf {X}}_{n} &{} {\mathbf {Z}}'_{n} {\mathbf {Z}}_{n} {\mathbf {M}}_{n} &{} {\mathbf {Z}}_{n}' {\mathbf {Z}}_{n}+ {\mathbf {A}}^{nn}\dfrac{\sigma _{e}^{2}}{\sigma _{g}^{2}} \end{array}\right] \left[ \begin{array}{c} \hat{\varvec{\beta }}\\ \hat{\varvec{\alpha }}\\ \hat{\varvec{\epsilon }} \end{array}\right] =\left[ \begin{array}{c} {\mathbf {X}}'{\mathbf {y}}\\ {\mathbf {M}}'{\mathbf {Z}}'{\mathbf {y}}\\ {\mathbf {Z}}_{n}'{\mathbf {y}}_{n} \end{array}\right] , \end{aligned}$$

where $${\mathbf {X}}=\left[ \begin{array}{c} {\mathbf {X}}_{n}\\ {\mathbf {X}}_{g} \end{array}\right] ,$$ $${\mathbf {Z}}=\left[ \begin{array}{cc} {\mathbf {Z}}_{n} &{} {\mathbf {0}}\\ {\mathbf {0}} &{} {\mathbf {Z}}_{g} \end{array}\right] ,$$ $${\mathbf {M}}=\left[ \begin{array}{c} {\mathbf {M}}_{n}\\ {\mathbf {M}}_{g} \end{array}\right] ,$$ $${\mathbf {y}}= \mathbf {\left[ \begin{array}{c} {\mathbf {y}}_{n}\\ {\mathbf {y}}_{g} \end{array}\right] },$$ $\sigma _{\alpha }^{2}$ is the variance of marker effects, $\sigma _{g}^{2}$ is the additive genetic variance, and $\sigma _{e}^{2}$ is the residual variance. These Eq. (3) contain matrix-by-matrix products and matrix-by-vector products involving the dense matrix ${\mathbf {M}}_{n}$ of imputed genotypes. We will assume here that ${\mathbf {X}}'{\mathbf {ZM}}, {\mathbf {M}}'{\mathbf {Z}}'{\mathbf {X}}$ and ${\mathbf {M}}'{\mathbf {Z}}'{\mathbf {ZM}}$ are small enough to be stored in memory. Below, we present computing strategies for calculations that involve ${\mathbf {Z}}'_{n}{\mathbf {Z}}_{n}{\mathbf {M}}_{n}$ or its transpose without storing these large matrices in memory. If the matrices, ${\mathbf {X}}'{\mathbf {ZM}}$ and ${\mathbf {M}}'{\mathbf {Z}}'{\mathbf {X}}$, are large, the computing strategies presented below can also be adapted for calculations that involve these matrices as will be done in our example application.

#### Computing strategies

First, we will discuss the calculations necessary to apply PCG to (3). Following this, we will discuss how to use (3) to obtain Markov chain Monte-Carlo (MCMC) samples of the location parameters of Model (1) from their full conditional distributions.

*Preconditioned conjugate gradient iteration* The PCG algorithm is widely used to iteratively solve the MME, e.g., [17, 18]. In each iteration of PCG, the left-hand-side of the MME (LHS-MME) is post-multiplied by a vector. However, the LHS-MME given in (3) contains two dense sub-matrices, ${\mathbf {Z}}'_{n}{\mathbf {Z}}_{n}{\mathbf {M}}_{n}$ and its transpose, that may be too large for storage in memory; the remaining sub-matrices in LHS-MME can be stored in memory either because they are not too large or because they are sparse. In each round of PCG, ${\mathbf {Z}}'_{n}{\mathbf {Z}}_{n}{\mathbf {M}}_{n}$ needs to be post-multiplied by a vector ${\mathbf {q}}$ that has the same order as $\varvec{\alpha }$ and the transpose of this matrix by a vector ${\mathbf {s}}$ that has the same order as $\varvec{\epsilon }$. The first of these products can be done without storing ${\mathbf {Z}}'_{n}{\mathbf {Z}}_{n}{\mathbf {M}}_{n}$ in memory as follows. Post-multiplying both sides of Eq. (2) by ${\mathbf {q}}$ gives:

4 $$\begin{aligned} {\mathbf {A}}^{nn}{\mathbf {M}}_{n}{\mathbf {q}} & = -{\mathbf {A}}^{ng}{\mathbf {M}}_{g}{\mathbf {q}}\nonumber \\ {\mathbf {A}}^{nn}{\mathbf {x}}& = {{\mathbf {b}}}, \end{aligned}$$

where ${\mathbf {x}}={\mathbf {M}}_{n}{\mathbf {q}}$ and ${{\mathbf {b}}}=-{\mathbf {A}}^{ng}({\mathbf {M}}_{g}{\mathbf {q}})$. Note that for efficient computation, the matrix ${\mathbf {M}}_{g}$ is first multiplied by ${\mathbf {q}}$ and the resulting vector is then premultiplied by the sparse matrix $-{\mathbf {A}}^{ng}$ to get ${{\mathbf {b}}}$. Solving the sparse system (4) gives the product ${\mathbf {x}}={\mathbf {M}}_{n}{\mathbf {q}}$ without storing the large dense matrix ${\mathbf {M}}_{n}$ in memory, and premultiplying ${\mathbf {x}}$ by ${\mathbf {Z}}'_{n}{\mathbf {Z}}_{n}$ gives the first product that is required for PCG. To obtain the second product, note that from Eq. (2),

5 $$\begin{aligned} {\mathbf {M}}_{n}'=-{\mathbf {M}}_{g}'{\mathbf {A}}^{gn}({\mathbf {A}}^{nn})^{-1}. \end{aligned}$$

Thus, the required product ${\mathbf {M}}_{n}'{\mathbf {Z}}'_{n}{\mathbf {Z}}_{n}{\mathbf {s}}$ can be written as $-{\mathbf {M}}_{g}'{\mathbf {A}}^{gn}({\mathbf {A}}^{nn})^{-1}{\mathbf {Z}}'_{n}{\mathbf {Z}}_{n}{\mathbf {s}}.$ To compute this efficiently, first the product ${{\mathbf {b}}}={\mathbf {Z}}'_{n}{\mathbf {Z}}_{n}{\mathbf {s}}$ is obtained. Then, solving the sparse system:

6 $$\begin{aligned} {\mathbf {A}}^{nn}{\mathbf {x}}={{\mathbf {b}}}, \end{aligned}$$

gives ${\mathbf {x}}=({\mathbf {A}}^{nn})^{-1}{\mathbf {Z}}'_{n}{\mathbf {Z}}_{n}{\mathbf {s}}$, where ${{\mathbf {b}}}$ and ${\mathbf {x}}$ have been reused to denote intermediate results in these computations. Next, ${\mathbf {x}}$ is premultiplied by the sparse matrix $-{\mathbf {A}}^{gn}$ and the resulting vector is premultiplied by ${\mathbf {M}}_{g}'$ to get the second product that is required for PCG. The remaining matrix-by-vector products for PCG can be obtained directly because these matrices are stored in memory.

We will now describe how these matrices and the right hand sides involving ${\mathbf {M}}_{n}$ can be computed in order to form the other elements of the MME without storing ${\mathbf {M}}_{n}$ in memory.

Consider computing:

$$\begin{aligned} {\mathbf {M}}'{\mathbf {Z}}'{\mathbf {ZM}} ={\mathbf {M}}'_{n}{\mathbf {Z}}'_{n}{\mathbf {Z}}_{n}{\mathbf {M}}_{n}+{\mathbf {M}}'_{g}{\mathbf {Z}}'_{g}{\mathbf {Z}}_{g}{\mathbf {M}}_{g}, \end{aligned}$$

without storing ${\mathbf {M}}_{n}$ in memory. Let $\mathbf {m}'_{n_{i}}$ denote row *i* of ${\mathbf {Z}}_{n}{\mathbf {M}}_{n}$. Then, ${\mathbf {M}}'_{n}{\mathbf {Z}}'_{n}{\mathbf {Z}}_{n}{\mathbf {M}}_{n}$ can be written as:

7 $$\begin{aligned} {\mathbf {M}}'_{n}{\mathbf {Z}}'_{n}{\mathbf {Z}}_{n}{\mathbf {M}}_{n}=\sum _{i}\mathbf {m}{}_{n_{i}}\mathbf {m}'_{n_{i}}. \end{aligned}$$

Thus, the matrix product ${\mathbf {M}}'_{n}{\mathbf {Z}}'_{n}{\mathbf {Z}}_{n}{\mathbf {M}}_{n}$ can be computed without storing ${\mathbf {M}}_{n}$ in memory if each row of ${\mathbf {Z}}_{n}{\mathbf {M}}_{n}$ can be obtained without computing the entire matrix. Rearranging (2), row *i* of ${\mathbf {Z}}_{n}{\mathbf {M}}_{n}$ can be computed as:

8 $$\begin{aligned} {\mathbf {m}}'_{n_{i}}=-\mathbf {e}'_{i}{\mathbf {Z}}_{n} ({\mathbf {A}}^{nn})^{-1}{\mathbf {A}}^{ng}{\mathbf {M}}_{g}, \end{aligned}$$

where ${\mathbf {e}}'_{i}$ is a row vector with 1 in the *i*th position and 0s elsewhere, and the product ${\mathbf {e}}'_{i}{\mathbf {Z}}_{n}({\mathbf {A}}^{nn})^{-1}$ can be obtained by solving the sparse system:

9 $$\begin{aligned} {\mathbf {A}}^{nn}{\mathbf {x}}={{\mathbf {b}}}, \end{aligned}$$

where ${{\mathbf {b}}}={\mathbf {Z}}'_{n}{\mathbf {e}}{}_{i}$. Note that the solution to (9) gives ${\mathbf {x}}'={\mathbf {e}}'_{i}{\mathbf {Z}}_{n}({\mathbf {A}}^{nn})^{-1}$, without having to invert ${\mathbf {A}}^{nn}$. These row vectors of ${\mathbf {Z}}_{n}{\mathbf {M}}_{n}$ can also be used to compute ${\mathbf {X}}'_{n}{\mathbf {Z}}_{n}{\mathbf {M}}_{n}$ as:

10 $$\begin{aligned} {\mathbf {X}}'_{n}{\mathbf {Z}}_{n}{\mathbf {M}}_{n}=\sum _{i}{\mathbf {x}}_{i}{\mathbf {m}}'_{n_{i}}, \end{aligned}$$

where ${\mathbf {x}}_{i}$ is used here to denote the *i*th column of ${\mathbf {X}}'_{n}$, which is the first term of

$$\begin{aligned} {\mathbf {X}}'{\mathbf {ZM}}={\mathbf {X}}'_{n}{\mathbf {Z}}_{n}{\mathbf {M}}_{n}+{\mathbf {X}}'_{g}{\mathbf {Z}}_{g}{\mathbf {M}}_{g}, \end{aligned}$$

which is a product of ${\mathbf {M}}_{n}$. Similarly, the right-hand-side vector ${\mathbf {M}}'{\mathbf {Z}}'{\mathbf {y}}$ can be written as the sum:

$$\begin{aligned} {\mathbf {M}}'{\mathbf {Z}}'{\mathbf {y}}={\mathbf {M}}'_{n}{\mathbf {Z}}'_{n}{\mathbf {y}}_{n}+{\mathbf {M}}'_{g}{\mathbf {Z}}'_{g}{\mathbf {y}}_{g}, \end{aligned}$$

which is a product of ${\mathbf {M}}_{n}$, and its first term can be computed as:

11 $$\begin{aligned} {\mathbf {M}}'_{n}{\mathbf {Z}}'_{n}{\mathbf {y}}_{n}=\sum _{i}{\mathbf {m}}_{n_{i}}y_{i}, \end{aligned}$$

where $y_{i}$ is used to denote the *i*th element of ${\mathbf {y}}_{n}.$

Note that computing ${\mathbf {m}}'_{n_{i}}$ corresponding to row *i* of ${\mathbf {Z}}_{n}{\mathbf {M}}_{n}$ using Eq. (8) can be done independently of its computation for any other row, and thus, the computations in Eqs. (7), (10), and (11) can be easily parallelized.

There are a number of approaches to compute ${\mathbf {m}}'_{n_i}$ that can be used. One approach for solving Eq. (9) for *n* less than approximately ten million using a typical workstation computer is to obtain a sparse Cholesky factor of ${\mathbf {A}}^{nn}$ and directly solve for each ${\mathbf {m}}'_{n_i}$ using forward and backward substitution. Software libraries exist for obtaining a Cholesky factor of large sparse matrices [19] using multiple threads or general purpose graphics processing units (GPU). Once the factor is obtained, independent threads can be used to solve in parallel from a single memory copy of the factor. Note that the Cholesky factor of ${\mathbf {A}}^{nn}$ may be denser than ${\mathbf {A}}^{nn}$, depending upon the nature of the relationships between genotyped and non-genotyped animals. For example, if non-genotyped animals comprise only non-parents, then ${\mathbf {A}}^{nn}$ is diagonal and solution for ${\mathbf {m}}_{i}'$ is trivial. For larger linear systems with non-genotyped parents where the Cholesky factor is too large, indirect solution using high performance methods on GPU is a practical alternative. The PCG algorithm parallelizes well and performs efficiently on GPU.

*MCMC sampling* Gibbs sampling is a widely used MCMC method for inference with Bayesian regression models, e.g., [3, 20, 21]. One of the most time-consuming tasks in these analyses is single-site sampling of the location parameters from their full-conditional distributions. Let $$\varvec{\theta }=\left[ \begin{array}{c} \varvec{\beta }\\ \varvec{\alpha }\\ \varvec{\epsilon } \end{array}\right]$$ denote the location parameters in Model (1). Then, following [22], the full-conditional distribution for $\theta _{i}$ under BayesC with $\pi =0$ is:

$$\begin{aligned} \theta _{i}|{\text {ELSE}} \sim {\text {N}}\left( \tilde{\theta }_{i},c_{ii}^{-1}\sigma _{e}^{2}\right) , \end{aligned}$$

where ELSE is used to denote all the other parameters in the model and the vector of phenotypes, $\tilde{\theta }_{i}$ is the solution to:

12 $$\begin{aligned} c_{ii}\tilde{\theta }_{i}=r_{i} -{\mathbf {c}}'_{i}\varvec{\theta }+c_{ii}\theta _{i}, \end{aligned}$$

$c_{ii}$ is the *i*th diagonal of the matrix ${\mathbf {C}}$ that denotes the LHS-MME given in (3), $r_{i}$ is the right-hand-side element from (3) corresponding to $\theta _{i}$, and $\mathbf {c}'_{i}$ is row *i* of ${\mathbf {C}}$. However, as mentioned previously, some sub-matrices of the LHS-MME given in (3) are dense and too large to be stored in memory. However, as explained below, the same strategy used to avoid storing these sub-matrices in PCG calculations can also be used here.

Consider computing the full conditional mean and variance for $\theta _{i}=\alpha _{j}$. Then $c_{ii}$, the *i*th diagonal element from ${\mathbf {C}}$ is obtained from the *j*th diagonal of ${\mathbf {B}}={\mathbf {M}}'{\mathbf {Z}}' {\mathbf {Z}}{\mathbf {M}}+\mathbf {I\dfrac{\sigma _{e}^{2}}{\sigma _{\alpha }^{2}}}$, which can be stored in memory. Similarly, $r_{i}-{\mathbf {c}}'_{i}\varvec{\theta }+c_{ii}\theta _{i}$ is computed as:

$$\begin{aligned} r_{i}-{\mathbf {c}}'_{i}\varvec{\theta }+c_{ii}\theta _{i}=d_{j}-\mathbf {b'}_{j}\varvec{\alpha }+b_{jj}\alpha _{j}, \end{aligned}$$

where $d_{j}$ is element *j* of the vector

$$\begin{aligned} {\mathbf {d}}={\mathbf {M}}'{\mathbf {Z}}'{\mathbf {y}}-{\mathbf {M}}'{\mathbf {Z}}'{\mathbf {X}}\varvec{\beta }-{\mathbf {M}}'_{n}{\mathbf {Z}}'_{n}{\mathbf {Z}}{}_{n}\varvec{\epsilon }, \end{aligned}$$

${{\mathbf {b}}}'_{j}$ is row *j* and $b_{jj}$ is the *j*th diagonal of ${\mathbf {B}}$. We have already seen how the large, dense matrix ${\mathbf {M}}_{n}'{\mathbf {Z}}'_{n}{\mathbf {Z}}_{n}$ can be multiplied by a vector such as $\varvec{\epsilon }$ without storing this matrix in memory, and this same strategy can be used here to compute ${\mathbf {M}}'_{n}{\mathbf {Z}}'_{n}{\mathbf {Z}}{}_{n}\varvec{\epsilon }$. The full-conditional distribution for $\alpha _{j}$, under BayesC with $\pi =0$, becomes:

$$\begin{aligned} \alpha _{j}|{\text {ELSE}} \sim {\text {N}}\left( \tilde{\alpha }_{j},b_{jj}^{-1}\sigma _{e}^{2}\right) , \end{aligned}$$

where $\tilde{\alpha }_{j}$ is the solution to:

13 $$\begin{aligned} b_{jj}\tilde{\alpha }_{j}=d_{j} -{\mathbf {b'}}_{j}\varvec{\alpha }+b_{jj}\alpha _{j}. \end{aligned}$$

The right-hand-side of this Eq. (13) is also used for calculations that involve variable selection in BayesB and BayesC when $\pi >0$ [21, 23].

Similarly, to compute full-conditional mean and variance for $\theta _{i}=\epsilon _{j}$, $c_{ii}$ is obtained from the *j*th diagonal of ${\mathbf {B}}$ that now denotes ${\mathbf {B}}={\mathbf {Z}}_{n}'{\mathbf {Z}}_{n}+{\mathbf {A}}^{nn}\dfrac{\sigma _{e}^{2}}{\sigma _{g}^{2}}$, and

$$\begin{aligned} r_{i}-{\mathbf {c}}'_{i}\varvec{\theta }+c_{ii}\theta _{i}=d_{j}-{\mathbf {b'}}_{j}\varvec{\epsilon }+b_{jj}\epsilon _{j}, \end{aligned}$$

where $d_{j}$ is element *j* of the vector that now denotes:

$$\begin{aligned} {\mathbf {d}}={\mathbf {Z}}_{n}'{\mathbf {y}}_{n} -{\mathbf {Z}}'_{n}{\mathbf {X}}_{n}\varvec{\beta } -{\mathbf {Z}}'_{n}{\mathbf {Z}}_{n}{\mathbf {M}}_{n}\varvec{\alpha }. \end{aligned}$$

The product ${\mathbf {Z}}'_{n}{\mathbf {Z}}_{n}{\mathbf {M}}_{n}\varvec{\alpha }$ is obtained as described for PCG calculations. Then, the full-conditional distribution for $\epsilon _{j}$ becomes:

$$\begin{aligned} \epsilon _{j}|{\text {ELSE}}\sim {\text {N}}\left( \tilde{\epsilon }_{j},b_{jj}^{-1}\sigma _{e}^{2}\right) , \end{aligned}$$

where $\tilde{\epsilon }_{j}$ is the solution to

$$\begin{aligned} b_{jj}\tilde{\epsilon }_{j}=d_{j} -{\mathbf {b'}}_{j}\varvec{\epsilon }+b_{jj}\epsilon _{j}. \end{aligned}$$

Samples of model effects such as $\varvec{\beta }, \varvec{\alpha }$, and $\varvec{\epsilon }$, or their linear functions that represent breeding values namely ${\mathbf {M}}_g\varvec{\alpha }$ for genotyped animals or ${\mathbf {M}}_n\varvec{\alpha } + \varvec{\epsilon }$ for non-genotyped animals can be accumulated as sums and sums of squares to obtain posterior means and prediction error variances. Alternatively, samples of fitted model effects can be written to a file for post-processing.

### Hybrid model for single-step Bayesian regression

The large, dense matrix ${\mathbf {M}}_{n}$ appears in the MEM given by Model (1). This is avoided here by using a BVM for animals with missing genotypes rather than expressing their breeding values as the sum of the effects of their imputed marker genotypes plus their separate imputation residuals. The advantages of the MEM such as allowing for alternative priors for marker effects are retained by still fitting a MEM but only for animals with genotypes. The hybrid model equation is:

14 $$\begin{aligned} \mathbf {\left[ \begin{array}{c} {\mathbf {y}}_{n}\\ {\mathbf {y}}_{g} \end{array}\right] }=\left[ \begin{array}{c} {\mathbf {X}}_{n}\\ {\mathbf {X}}_{g} \end{array}\right] \varvec{\beta }+\left[ \begin{array}{cc} {\mathbf {0}} &{} {\mathbf {Z}}_{n}\\ {\mathbf {Z}}_{g}{\mathbf {M}}_{g} &{} {\mathbf {0}} \end{array}\right] \left[ \begin{array}{l} \varvec{\alpha }\\ {\mathbf {u}}_{n} \end{array}\right] +{\mathbf {e}}, \end{aligned}$$

with ${\mathbf {u}}_{n}={\mathbf {M}}_{n}\varvec{\alpha }+\varvec{\epsilon }$, and thus, this single-step hybrid model (SS-HM) is equivalent to the SS-MEM (1). To construct the MME for this model (14), we need to invert the covariance matrix corresponding to the random effects, namely $$\varvec{\Sigma }={\text {Var}}\left( \left[ \begin{array}{l} \varvec{\alpha }\\ {\mathbf {u}}_{n} \end{array}\right] \right)$$. That inverse can be obtained by first writing the random effects of (14) as:

$$\begin{aligned} \left[ \begin{array}{l} \varvec{\alpha }\\ {\mathbf {u}}_{n} \end{array}\right] =\left[ \begin{array}{cc} {\mathbf {I}} &{} {\mathbf {0}}\\ {\mathbf {M}}_{n}&{} {\mathbf {I}} \end{array}\right] \left[ \begin{array}{l} \varvec{\alpha }\\ \varvec{\epsilon } \end{array}\right] . \end{aligned}$$

Then, $\varvec{\Sigma }$ can be written as:

$$\begin{aligned} \varvec{\Sigma } & = {} \left[ \begin{array}{cc} {\mathbf {I}} &{} {\mathbf {0}}\\ {\mathbf {M}}_n &{} {\mathbf {I}} \end{array}\right] {\text {Var}}\left( \left[ \begin{array}{l} \varvec{\alpha }\\ \varvec{\epsilon } \end{array}\right] \right) \left[ \begin{array}{cc} {\mathbf {I}} &{} {\mathbf {M}}'_{n}\\ {\mathbf {0}} &{} {\mathbf {I}} \end{array}\right] \\ & = {} \left[ \begin{array}{cc} {\mathbf {I}} &{} {\mathbf {0}}\\ \mathbf {{\mathbf {M}}_{n}} &{} {\mathbf {I}} \end{array}\right] \left[ \begin{array}{cc} {\mathbf {I}}\sigma _{\alpha }^{2} &{} {\mathbf {0}}\\ {\mathbf {0} }&{} ({\mathbf {A}}^{nn})^{-1}\sigma _{g}^{2} \end{array}\right] \left[ \begin{array}{cc} {\mathbf {I}} &{} {\mathbf {M}}'_{n}\\ {\mathbf {0}} &{} {\mathbf {I}} \end{array}\right] , \end{aligned}$$

and its inverse can be obtained as:

$$\begin{aligned} \varvec{\Sigma }^{-1} & = {} \left[ \begin{array}{cc} {\mathbf {I}} &{} -{\mathbf {M}}'_{n}\\ {\mathbf {0}} &{} {\mathbf {I}} \end{array}\right] \left[ \begin{array}{cc} {\mathbf {I}}\frac{1}{\sigma _{\alpha }^{2}} &{} {\mathbf {0}}\\ {\mathbf {0}} &{} {\mathbf {A}}^{nn}\frac{1}{\sigma _{g}^{2}} \end{array}\right] \left[ \begin{array}{cc} {\mathbf {I}} &{} {\mathbf {0}}\\ -{\mathbf {M}}_{n} &{} {\mathbf {I}} \end{array}\right] \\ & = {} \left[ \begin{array}{cc} {\mathbf {I}} &{} {\mathbf {0}}\\ {\mathbf {0}} &{} {\mathbf {0}} \end{array}\right] \frac{1}{\sigma _{\alpha }^{2}}+\left[ \begin{array}{c} -{\mathbf {M}}'_{n}\\ {\mathbf {I}} \end{array}\right] {\mathbf {A}}^{nn}\frac{1}{\sigma _{g}^{2}}\left[ \begin{array}{cc} -{\mathbf {M}}_{n}&{\mathbf {I}}\end{array}\right] \\ & = {} \left[ \begin{array}{cc} {\mathbf {I}} &{} {\mathbf {0}}\\ {\mathbf {0}} &{} {\mathbf {0}} \end{array}\right] \frac{1}{\sigma _{\alpha }^{2}}+ \left[ \begin{array}{cc} {\mathbf {M}}'_{n}{\mathbf {A}}^{nn}{\mathbf {M}}_{n} &{} -{\mathbf {M}}'_{n}{\mathbf {A}}^{nn}\\ -{\mathbf {A}}^{nn}{\mathbf {M}}_{n} &{} {\mathbf {A}}^{nn} \end{array}\right] \frac{1}{\sigma _{g}^{2}}. \end{aligned}$$

Now, using the result ${\mathbf {M}}_{n}={\mathbf {A}}_{ng}{\mathbf {A}}_{gg}^{-1}{\mathbf {M}}_{g}=-({\mathbf {A}}^{nn})^{-1}{\mathbf {A}}^{ng}{\mathbf {M}}_{g}$ [10], in the off-diagonal blocks of the second term, $\varvec{\Sigma ^{-1}}$ becomes:

$$\begin{aligned} \varvec{\Sigma }^{-1}=\left[ \begin{array}{cc} {\mathbf {I}}\frac{1}{\sigma _{\alpha }^{2}}+{\mathbf {M}}'_{n} {\mathbf {A}}^{nn}{\mathbf {M}}_{n}\frac{1}{\sigma _{g}^{2}} &{} {\mathbf {M}}'_{g}{\mathbf {A}}^{gn}\frac{1}{\sigma _{g}^{2}}\\ {\mathbf {A}}^{ng}{\mathbf {M}}_{g}\frac{1}{\sigma _{g}^{2}} &{} {\mathbf {A}}^{nn}\frac{1}{\sigma _{g}^{2}} \end{array}\right] , \end{aligned}$$

and then the MME for the HM (14) can be written as:

15 $$\begin{aligned} \left[ \begin{array}{lll} {\mathbf {X}}'{\mathbf {X}} &{} {\mathbf {X}}'_{g}{\mathbf {Z}}_{g}{\mathbf {M}}_{g} &{} {\mathbf {X}}_{n}'{\mathbf {Z}}_{n}\\ {\mathbf {M}}'_{g}{\mathbf {Z}}'_{g}{\mathbf {X}}_{g} &{} \mathbf {Q} &{} {\mathbf {M}}'_{g}{\mathbf {A}}^{gn}\frac{\sigma _{e}^{2}}{\sigma _{g}^{2}}\\ {\mathbf {Z}}'_{n}{\mathbf {X}}_{n} &{} {\mathbf {A}}^{ng}{\mathbf {M}}_{g}\frac{\sigma _{e}^{2}}{\sigma _{g}^{2}} &{} {\mathbf {Z}}_{n}'{\mathbf {Z}}_{n}+{\mathbf {A}}^{nn}\dfrac{\sigma _{e}^{2}}{\sigma _{g}^{2}} \end{array}\right] \left[ \begin{array}{c} \hat{\varvec{\beta }}\\ \hat{\varvec{\alpha }}\\ \hat{\mathbf {u}}_{n} \end{array}\right] =\left[ \begin{array}{c} {\mathbf {X}}'{\mathbf {y}}\\ {\mathbf {M}}'_{g}{\mathbf {Z}}'_{g}{\mathbf {y}}_{g}\\ {\mathbf {Z}}_{n}'{\mathbf {y}}_{n} \end{array}\right] , \end{aligned}$$

where ${\mathbf {Q}} = {\mathbf {M}}'_{g}{\mathbf {Z}}'_{g}{\mathbf {Z}}_{g}{\mathbf {M}}_{g}+ {\mathbf {I}}\frac{\sigma _{e}^{2}}{\sigma _{\alpha }^{2}}+ {\mathbf {M}}'_{n}{\mathbf {A}}^{nn}{\mathbf {M}}_{n}\frac{\sigma _{e}^{2}}{\sigma _{g}^{2}}.$ These equations involve ${\mathbf {M}}_{g}$ rather than ${\mathbf {M}}_{n}$, except in $\mathbf {Q}$, the diagonal block that corresponds to $\hat{\varvec{\alpha }}$, which has dimension equal to the number of marker covariates, often less than 50,000, regardless of the number of genotyped or non-genotyped animals. Furthermore, we assume here that ${\mathbf {X}}'_{g}{\mathbf {Z}}_{g}{\mathbf {M}}_{g}$ and ${\mathbf {M}}'_{g}{\mathbf {Z}}'_{g}{\mathbf {X}}_{g}$ are small enough to be stored in memory.

The only difference between Eq. (15) and the MME given by Equation (A1) in Legarra and Ducrocq [15] is in ${\mathbf {Q}}$. Using the notation in this paper, the matrix expression ${\mathbf {M}}'_{n}{\mathbf {A}}^{nn}{\mathbf {M}}_{n}$ that is present in the ${\mathbf {Q}}$ is expressed as ${\mathbf {M}}'_{g}({\mathbf {A}}^{gg} - {\mathbf {A}}^{-1}_{gg}){\mathbf {M}}_{g}$ in that paper [15], which involves the inverse of ${\mathbf {A}}_{gg}$ that is difficult to compute. However, these two expressions are identical because $({\mathbf {A}}^{gg} - {\mathbf {A}}_{gg}^{-1}) = {\mathbf {A}}^{gn} ({\mathbf {A}}^{nn})^{-1} {\mathbf {A}}^{ng}$ and ${\mathbf {M}}_n = -({\mathbf {A}}^{nn})^{-1} {\mathbf {A}}^{ng} {\mathbf {M}}_g$.

#### Computing strategies 

The matrix ${\mathbf {M}}_{n}$ of imputed genotypes does not appear alone in the MME (15), but the MME involve rather the matrix product ${\mathbf {M}}'_{n}{\mathbf {A}}^{nn}{\mathbf {M}}_{n}$. However, ${\mathbf {M}}'_{n}{\mathbf {A}}^{nn}{\mathbf {M}}_{n}$ can be computed efficiently without needing to store the entire ${\mathbf {M}}_{n}$ matrix in memory, in situations when the number of genotyped animals is less than the number of non-genotyped animals. To do so, first from Eq. (5) ${\mathbf {M}}'_{n}{\mathbf {A}}^{nn}=-{\mathbf {M}}'_{g}{\mathbf {A}}^{gn}$. Next, column *i* of ${\mathbf {M}}_{n}$ is obtained by solving the sparse system (2) for column *i* and premultiply it by the sparse matrix ${\mathbf {A}}^{gn}$. This gives column *i* of the product ${\mathbf {A}}^{gn}{\mathbf {M}}_{n}$, which has the same size as ${\mathbf {M}}_{g}$. The columns of ${\mathbf {A}}^{gn}{\mathbf {M}}_{n}$ can be computed one at a time or in parallel. Premultiplying ${\mathbf {A}}^{gn}{\mathbf {M}}_{n}$ by $-{\mathbf {M}}'_{g}$ gives:

16 $$\begin{aligned} -{\mathbf {M}}'_{g}{\mathbf {A}}^{gn}{\mathbf {M}}_{n} ={\mathbf {M}}'_{n}{\mathbf {A}}^{nn}{\mathbf {M}}_{n}. \end{aligned}$$

This needs to be done only once to set up the MME and has order equal to the number of marker genotypes which is often much less than the number of genotyped or non-genotyped animals.

These MME also contain two large, dense sub-matrices, namely ${\mathbf {A}}^{ng}{\mathbf {M}}_{g}$ and its transpose. As described previously, in the PCG iteration and in the Gibbs sampling, these matrices need to be post-multiplied by a vector. When the number of genotyped animals is sufficiently smaller than the number of non-genotyped animals, these matrix-by-vector products can be obtained more efficiently by storing in memory the sparse matrix ${\mathbf {A}}^{ng}$ and the dense but smaller matrix ${\mathbf {M}}_{g}$ rather than their product ${\mathbf {A}}^{ng}{\mathbf {M}}_{g}$. In each round of PCG iteration or Gibbs sampling, the matrix-by-vector product ${\mathbf {A}}^{ng}{\mathbf {M}}_{g}{\mathbf {q}}$, for example, is obtained by first multiplying the dense matrix ${\mathbf {M}}_{g}$ by the vector ${\mathbf {q}}$ and then premultiplying the result by the sparse matrix ${\mathbf {A}}^{ng}$. The corresponding calculation for the MEM required solving sparse systems of equations given by Eq. (4) in each round of PCG or Gibbs sampling, in addition to the two matrix-by-vector multiplications that are also required here.

In situations when the number of genotyped animals exceeds the number of non-genotyped animals, using SS-MEM that explicitly involves ${\mathbf {M}}_{n}$ in off-diagonal blocks may be competitive with SS-HM.

## Application of hybrid model

An example dataset from the American Simmental Association is used to demonstrate the computing effort to obtain PCG samples from (15) and the relative computing effort to obtain MCMC samples for the MME of (15) compared to (3). The vector of phenotypes comprised of 4,934,101 birth weight observations; there were 6,179,960 animals in the pedigree file; 31,453 animals in the pedigree file were genotyped and 23,290 of those had birth weight observations. After filtering marker covariates for low minor allele frequency, 40,214 marker effects were included in the model. There were 399,036 fixed effects, including the herd-year-season effects defined in the same manner as in the routine national evaluation. To keep this presentation that compares the computational effort involved in fitting (3) and (15) simple, our application was limited to a single trait ignoring maternal genetic and permanent environmental effects. Furthermore, we did not include a comparison with SS-GBLUP since that model cannot accommodate mixture priors for marker effects as used in this example.

The analyses were performed using a workstation built on an ASUS X99E WS motherboard, a Xeon E5-1650V3 3.5 Ghz processor overclocked to 4.2 Ghz, 128 GB of DDR4 ECC RAM at 2133 Mhz and four NVidia Titan X GPU, with 9TB of workspace in a RAID5 configuration comprising four SATA disks. The operating system was Ubuntu 14.04 LTS, and the BOLT software package (http://manual.thetasolutionsllc.com/IntroBolt) built with the CUDA Toolkit 7.5 was used.

The vector ${\mathbf {y}}$, and matrices ${\mathbf {X}},{\mathbf {Z}},{\mathbf {A}}^{nn},{\mathbf {A}}^{gn},{\mathbf {A}}^{ng},$ and ${\mathbf {M}}_{g}$ were built from data files using BOLT tools. Ordering the pedigree file, construction of ${\mathbf {A}}^{-1}$, including calculation of inbreeding, and its partitioning into blocks representing genotyped and non-genotyped animals took 3 min and required 1.0 Gb of disk storage and 302 Mb of memory. While ${\mathbf {A}}^{-1}$ was being formed, ${\mathbf {y}}$, ${\mathbf {X}}$ and ${\mathbf {Z}}$ were created in about 10 s and required 38, 78 and 83 Mb of disk storage. When stored in memory, they required 19.7, 59.2 and 59.2 Mb respectively. The matrix ${\mathbf {M}}_{g}$ required 4.2 Gb of disk and memory when stored in single precision.

The matrix product ${\mathbf {X}}'_{g}{\mathbf {Z}}_{g}{\mathbf {M}}_{g}$ and its transpose ${\mathbf {M}}'_{g}{\mathbf {Z}}'_{g}{\mathbf {X}}_{g}$ were not explicitly formed, instead computations involving those terms were done in parts as described previously in this paper. The sparse Cholesky decomposition of the 6,148,507 order $\mathbf {A}^{nn}$ matrix took just under 4 min. The imputation of ${\mathbf {M}}_{n}$, using forward and backward substitution with the Cholesky factor, and its premultiplication by ${\mathbf {A}}^{gn}$ took just over 35 min using eight parallel processes. The creation of the matrix products ${\mathbf {M}}'_{g}{\mathbf {Z}}'_{g}{\mathbf {Z}}_{g}{\mathbf {M}}_{g}$ and ${\mathbf {M}}'_{n}{\mathbf {A}}^{nn}{\mathbf {M}}_{n}$ each took about 20 s using 2 GPU after obtaining the imputed values and required 6.2 Gb of disk storage and memory when stored in single precision.

For the analysis using Eq. (15) the PCG solution of the MME stored in double precision took just under 40 min, using a single GPU and diagonal preconditioning. Because the PCG was performed in double precision just under 18 Gb of memory was required to store all the sub-matrices comprising the left-hand side. The right-hand side required 53Mb of memory. Additional memory for work space of approximately 4 Gb was required for PCG. Convergence was determined by comparing solutions from every 200 rounds of iteration to solutions from 5000 rounds. By 1800 rounds the correlation and regression of solutions with those from 5000 rounds were very close to one (.99 each). The PCG residual value was near 1.1e$-05$. The PCG solution does not give the posterior mean of the marker effects for a model with mixture priors, but was used to define starting values for MCMC sampling of all the effects in the MME, but using a mixture prior for marker effects.

Starting with the same PCG solution but different random number generator seed values, using 4 parallel chains each drawing 10,500 samples on its own GPU took 70 min to obtain a total of 42,000 Gibbs samples, using $\pi =0.95$ and known variance ratios in Eqs. (3) and (15). Each of the parallel Gibbs Sampler jobs shared a single copy in shared memory of the left-hand-side matrices, reducing the memory requirements and reading from disk.

Experience has shown that 40,000 samples after burn-in is sufficient to obtain posterior means of breeding values that are stable for the hybrid model and MEM. However, we confirmed this by sampling four additional parallel chains each with length 250,000 samples after a 5000 sample burn. The purpose of these very long chains was to confirm that the 40,000 length post-burn-in chain was sufficient, thus supporting the timings provided here to achieve useful results. The correlation of the posterior means of the breeding values for genotyped animals and non-genotyped animals from the aggregated 40,000 length chain was .99 and 1.0, respectively, with the posterior means from the aggregated 1,000,000 length chain. However, a chain longer that 40,000 may be needed to accurately estimate PEV for animals with intermediate to low accuracies. Because only off-diagonal blocks of the left-hand side are used in the GPU computation for updating the right-hand-sides for each vector of single-site Gibbs samples for $\varvec{\beta }$, $\varvec{\alpha }$ or ${\mathbf {u}}_{n}$, and the Gibbs samples were obtained using single precision, the entire left-hand side without the diagonal blocks fit on the GPU. This strategy also allowed the GPU to asynchronously update the right-hand-side while the next set of effects was being sampled using the CPU.

The Gibbs sampler was performed using single precision for storage of the left- and right-hand sides, requiring approximately half as much memory as the 18 Gb required for the PCG which was performed in double precision. Additional memory for work space of approximately 2 Gb was required for the Gibbs sampler. The total time required to assemble the left- and right-hand sides, after the matrix components were formed, was just under 3 min. The total job time for all steps, starting with the raw data, to obtain posterior mean estimates of the MCMC samples of marker effects and MCMC samples of breeding values of the genotyped and non-genotyped animals and their prediction error variances (from the posterior variances of their MCMC samples), took approximately 3 h.

The memory required to store ${\mathbf {M}}_{g}$ is determined by the product of the number of animals genotyped and the number of marker covariates. A compressed dense format (CBRC) allows this matrix to be 32 times larger than with the double precision version used above, but increased the computing time for PCG in this example by 25%.

An additional Gibbs sampler run was made with the MME of (3) that used Eq. (4), which requires within each iteration, forward and backward solves using the factor of ${\mathbf {A}}^{nn}$. The time required to obtain one sample of all effects was 2.0 s. Using the MME of (15) required only 0.44 s for each sample of all effects. Accordingly, the hybrid model has considerable advantage over that of [10]. These two computing approaches should give the same estimates of breeding values as they represent equivalent models as explained in the theory section. The correlations between the MCMC-derived estimates of breeding values between the two approaches were 1.0 for non-genotyped animals and over 0.99 for genotyped animals.

Computational performance of Eq. (15) was compared ignoring the genotypes on approximately half the genotyped animals to demonstrate the effect of the proportion of genotyped animals on computing time. This reduced dataset left 15,694 animals with genotype information of which 11,683 had a birth weight observation. The total number of animals in the pedigree file and number of observations on birth weight were the same as before. After filtering the marker covariates for low minor allele frequency, 40,211 marker loci remained. The time necessary to complete the PCG solver was about 3 min less than the 27 min needed for the larger analysis, which had approximately double the number of genotyped animals. The reduction in time necessary to complete the PCG solver was primarily due to reductions in time used for matrix multiplications involving the smaller matrix ${\mathbf {M}}_{g}$. The time necessary to obtain the 42,000 Gibbs samples was reduced by about 20% to 1 h. Imputation required 24 instead of 35 min. Creating ${\mathbf {M}}'_{g}{\mathbf {Z}}'_{g}{\mathbf {Z}}_{g}{\mathbf {M}}_{g}$ and ${\mathbf {M}}'_{n}{\mathbf {A}}^{nn}{\mathbf {M}}_{n}$ required just under 20 s, the same as before. Thus, doubling the proportion of genotyped animals increased the total job time from about 2.5 to 3 h.

## Discussion

Fernando et al. [10] introduced a single-step MEM that is equivalent to SS-GBLUP in the special case when all markers are fitted in the model. It has the advantage compared to SS-GBLUP that it can accommodate a wider class of models with different priors including mixture distributions. However, the MME corresponding to that model includes large, dense off-diagonal sub-matrices, ${\mathbf {Z}}'_{n}{\mathbf {Z}}_{n}{\mathbf {M}}_{n}$ and its transpose, between the blocks for marker effects and the imputation residuals. These sub-matrices are prohibitively large from a storage and computational viewpoint when there is a large number of non-genotyped animals. We have shown here that these limitations can be circumvented by representing those sub-matrices as:

$$\begin{aligned} -{\mathbf {Z}}'_{n}{\mathbf {Z}}_{n}({\mathbf {A}}^{nn})^{-1}{\mathbf {A}}^{ng}{\mathbf {M}}_{g} \end{aligned}$$

and its transpose, and by doing matrix multiplication in parts. This is possible for large problems but requires repeated solutions of an equation of the form ${\mathbf {A}}^{nn}{\mathbf {x}}={{\mathbf {b}}}.$ A similar solution is used in every iteration of SS-GBLUP when an APY inverse is exploited [18]. Nevertheless, the model in [10] is practical for realistic problems as demonstrated. It does not require approximations [18] as in SS-GBLUP when large numbers of animals are genotyped.

Here we have introduced a single-step HM that is equivalent to the MEM in [10] and in a special case equivalent to SS-GBLUP. The HM includes marker effects and breeding values, and the off-diagonal sub matrices comprise the term ${\mathbf {A}}^{ng}{\mathbf {M}}_{g}.$ Computations that involve this sub-matrix can be done efficiently in parts without having to solve equations of the form ${\mathbf {A}}^{nn}{\mathbf {x}}={{\mathbf {b}}}.$

The off-diagonal sub-matrices in both these models are the same size, the lower off-diagonal matrix being of the order of the number of non-genotyped animals by the number of markers. In SS-HM, this sub-matrix has a more convenient structure for storage and computation than is generally the case for SS-MEM. We have demonstrated its practicality and its considerable reduction in computing effort. This model can be readily extended to accommodate multiple traits, multiple breeds, maternal effects, and additional random effects such as polygenic residual effects.
